# An experimental protocol for mimicking pathomechanisms of traumatic brain injury in mice

**DOI:** 10.1186/2040-7378-4-1

**Published:** 2012-02-02

**Authors:** Christiane Albert-Weißenberger, Csanád Várrallyay, Furat Raslan, Christoph Kleinschnitz, Anna-Leena Sirén

**Affiliations:** 1 University of Würzburg, Department of Neurology, Würzburg, Germany; 2 University of Würzburg, Department of Neuroradiology Würzburg, Germany; 3 University of Würzburg, Department of Neurosurgery, Würzburg, Germany

**Keywords:** closed head injury, traumatic brain injury, neurobehavioural deficits, astrocyte, microglia, neurons

## Abstract

Traumatic brain injury (TBI) is a result of an outside force causing immediate mechanical disruption of brain tissue and delayed pathogenic events. In order to examine injury processes associated with TBI, a number of rodent models to induce brain trauma have been described. However, none of these models covers the entire spectrum of events that might occur in TBI. Here we provide a thorough methodological description of a straightforward closed head weight drop mouse model to assess brain injuries close to the clinical conditions of human TBI.

## 1 Introduction

Traumatic brain injury (TBI) is a result of an outside force causing immediate mechanical disruption of brain tissue and delayed pathogenic events that can exacerbate the injury (reviewed by [1]). It represents a leading cause of death and disability in the industrialized countries [2,3] and a growing health problem in the developing countries [4-7]. To better understand the pathological mechanisms underlying TBI and to develop strategies and interventions to limit the secondary damage, the use of rodent models is essential. A number of rodent models to induce brain trauma have been described; however, none of them covers the entire spectrum of events that might occur in TBI [8]. As an example, the cortical cryolesion model is particularly suited for investigating TBI-associated focal lesions with blood-brain barrier leakage and vasogenic brain edema [9-13] but contre coup and diffuse axonal injuries that typically complicate human head injuries [1] are missing. This pathophysiology is present in the weight drop models of TBI which use the gravitational forces of a free falling weight to produce a mix of focal and diffuse brain injury [14-16]. One main characteristic of TBI associated diffuse axonal injury is the axonal disruption caused by shearing forces. Typical pathological changes include axonal swelling, axoplasmic ovoid retraction balls and expression of amyloid beta peptides [17]. The original weight drop model in rats by Feeney [18,19] was optimimized to produce an open-head brain injury whereas the model developed by Shohami [20-22] produces a closed-head brain injury with both focal and diffuse injury and enables its adaptation for mice. We provide here a methodological description of our modification of the Shohami model [14,21] to induce closed head weight drop injury in mice.

## 2 Methods

All experiments require an appropriate animal experimentation facility and need to be conducted in accordance with the laws and regulations of the regulatory authorities for animal care. The animal experiments presented here were approved by and conducted in accordance with the laws and regulations of the regulatory authorities for animal care and use in Lower Franconia. A total of 20 male C57BL/mice (10-16 weeks) with a body weight of > 20 g mice were used.

### 2.1 Weight drop model - Surgery

**Figure 1:**
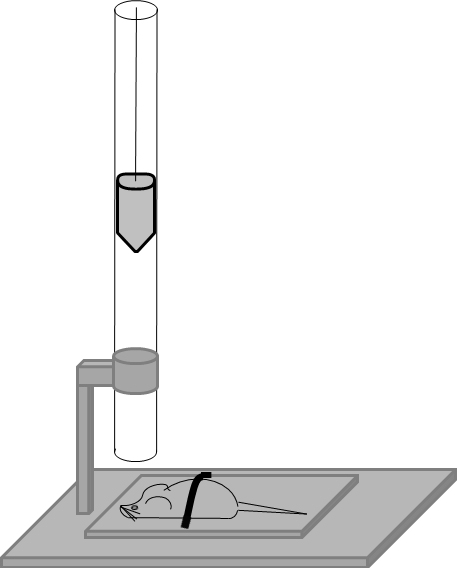
**Weight drop injury device**. The falling height of the free-falling weight with a silicon-covered blunt tip of 2 mm diameter can be carefully determined by a locking mechanism. Releasing a weight of 75 g from a height of 10 cm will cause a final impact of 0.01 J.

**Figure 2:**
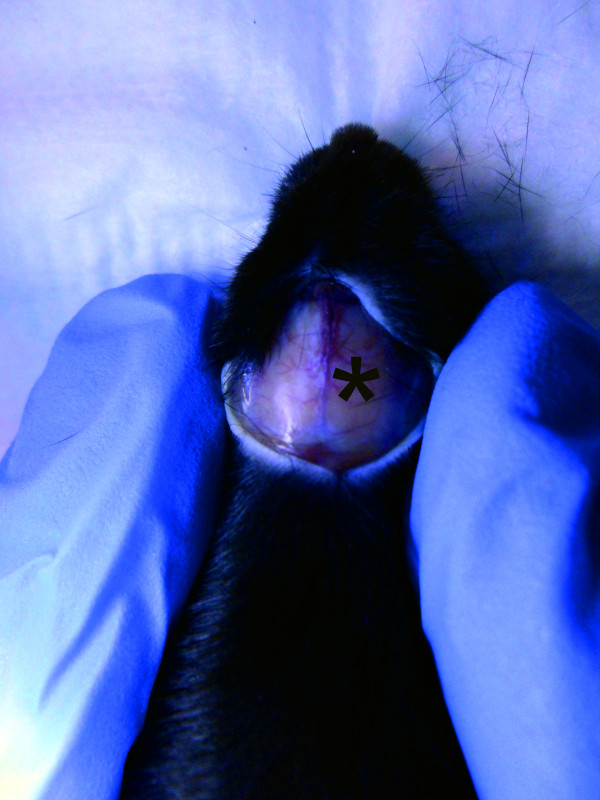
**Target area for rod impact**. The correct area to be impacted should lie on the right hemisphere and is identified by the asterisk.

After induction of anaesthesia with 4% isoflurane, a mouse was placed onto the platform directly under the weight of the weight drop device (Figure 1). Anaesthesia was maintained by mask inhalation of isoflurane vaporized at concentrations of 1.5% during the surgical procedures and depth of anaesthesia was monitored by toe pinch using tweezers. A midline longitudinal scalp incision was made and the skull exposed. After identification of the right anterior frontal area (1.5 mm lateral to the midline in the mid-coronal plane) as impact area (Figure 2), the weight was released and dropped with a final impact of 0.01 J onto the skull.

As the impact can result in trauma-induced respiratory depression and death, posttraumatic oxygen was immediately applied. Then the scalp wound was closed by standard suture material (3-0 Ethilon) and the wound area was treated with lidocain cream. Mice were returned to cages immediately at the end of the surgical procedures where access to water and food is freely available.

### 2.2 Outcome

Key readout parameters include the assessment of neurobehavioral outcome, activation of resident cells, neurodegeneration, and morphological changes.

#### 2.2.1 Neurobehavioral outcome

During the last years, an extensive number of studies have been accomplished showing that human TBI are associated with physical and cognitive deficits (for a recent review see [23]). In parallel to human TBI, the brain injury caused by the weight drop model presented here is also associated with neurobehavioral deficits [21]. The neurobehavioral status of mice was obtained by the neurological severity score (NSS) (adapted from [14]). It consists of 10 individual clinical parameters, including tasks on motor function, alertness and physiological behaviour. Each parameter is described below.

##### A) Exit circle

**Figure 3:**
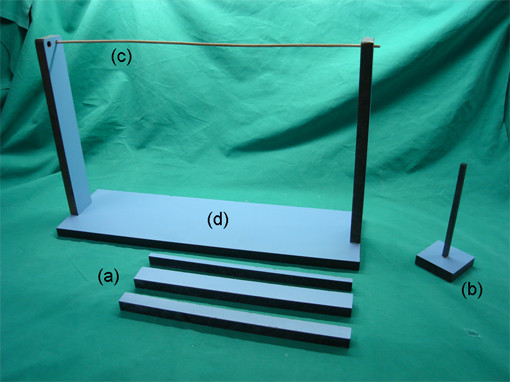
**Depiction of the equipment used for assessment of the NSS**. (a) Beam walk test. The healthy mouse will spontaneously cross 3-, 2- and 1-cm beams. The beam tests should start with the 3-cm beam and increase the level of difficulty by gradually reducing the beam width to 2 and 1 cm. (b) Beam for the beam balancing test. (c) The round stick balance test represents the most difficult test for mice. (d) The platform is used to evaluate the exploring behavior. Owing to their intrinsic inquisitiveness, a healthy mouse exits the platform within 2 min.

The mouse was placed in the middle of a platform (Figure 3d) and monitored how long it takes the mouse to exit the platform. Owing to their intrinsic seeking behaviour, healthy mice usually exited the platform within 2 min (0 points). If the mouse failed to exit the platform within 2 min there was given 1 point.

##### B) Seeking behavior

The mouse was placed on the platform and the exploring of the environment and sniffing behavior was monitored. While healthy mice explored the environment (0 points), injured mice do not display this physiological behaviour (1 point).

##### C) Monoparesis/hemiparesis

Monoparesis or hemiparesis was represented by failure to use one (monoparesis) or two (hemiparesis) paws. When being picked up by the tail, a healthy mouse intrinsically gripped a small forceps touching its paws and holds on to it (0 points). If the mouse failed to grip there was given 1 point.

##### D) Straight walk

The mouse was placed on an even surface and alertness, initiative and motor ability to walk straight was assessed. If a mouse failed to walk straight due to missing initiative or dragging of one or several paws 1 point was given.

##### E) Startle reflex

A healthy mouse reacted with a bounce and/or wince to a sound stimulus (hand clapping) (score of 0). If the mouse failed to respond 1 point was given.

##### F) Beam balancing

The mouse was placed on a beam of 7 mm × 7 mm (Figure 3b). Healthy mice are able to balance on the beam for at least 10 s. If a mouse failed to balance, 1 point was given.

##### G) Beam walk

Motor coordination and balance was also evaluated by the ability of a mouse to transverse a graded series of beams (Figure 3a). If a mouse failed to cross the 3-cm-wide and 30-cm-long beam, 3 points were scored and the test stopped. If a mouse managed to cross the 3-cm beam, then the test was repeated using a beam with a width of 2 cm. For a mouse failing to cross the 2-cm- beam, 2 points were scored and the test stopped. If a mouse managed to cross the 3-cm and 2-cm beam, but failed to cross the 1-cm beam, 1 point was given. Healthy mice managed to cross all beams (0 points).

##### H) Round stick balancing

**Figure 4:**
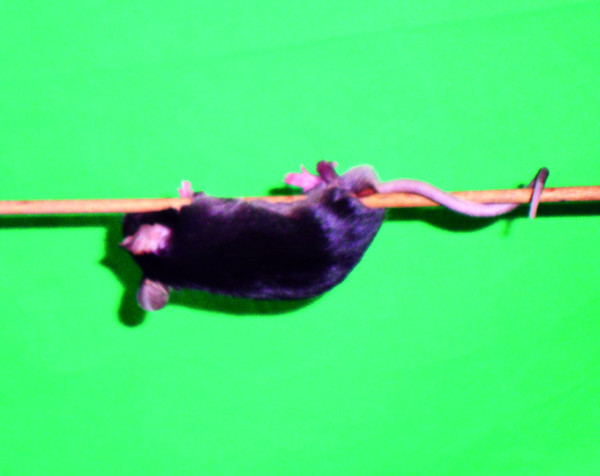
**Round stick balance test evaluation**.

Balance and grip strength are required to hold on a round stick of 3 mm diameter (Figure 3c and 4). If a mouse failed to hang onto string by at least two paws 1 point was given.

**Figure 5:**
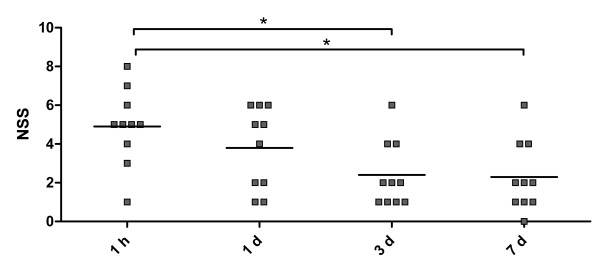
**The assessment of the NSS after trauma allows evaluation of the recovery**. Initial severity of the trauma was assessed 1 h after trauma (group mean of 4.90 +- 0.62, n = 10). Compared to the initial score value 1 h after trauma, the NSS values were significantly decreased at day 3 and 7 (*P < 0.05).

The points of each task were summed up to obtain the NSS. Initial severity of the trauma was assessed 1 h after trauma. All animals in this study exhibited neurological deficits 1 h after trauma, with a group mean of 4.90 +- 0.62 (n = 10) (Figure 5). Evaluation of the NSS values at later time points revealed that the neurological status improved over time and compared to the initial score value 1 h after trauma, the NSS values were significantly decreased at day 3 and 7 (n = 10, P < 0.05). These results show that the NSS is an excellent tool to estimate the initial severity of and recovery from brain injury induced by weight drop. Sham operated mice possessed no neurobehavioral deficits, reflected by a score of 0. In order to avoid bias in interpretation of the results evaluation of task performance were carried out in a blinded manner.

#### 2.2.2 Immunhistochemical characterization

Studies characterising the posttraumatic response of resident brain cells in humans, show that TBI leads to activation of astrocytes, microglia, and neurodegeneration [24-27]. Similarly, brain injury induced by the weight drop model presented here initiates pathological processes leading to cellular changes in glia and neurons.

To detect activation of glia immunohistochemical stainings were performed as previously described [28]. Briefly, 10-μm thin brain cryosections were prepared using a cryostat (Leica, Bensheim, Germany). The immunohistochemical staining with antibodies for the detection of glial fibrillary acidic protein (GFAP)-expressing astrocytes (rabbit, 1:500, anti-GFAP; Ab7260; Abcam, Cambridge, UK) and activated microglia/macrophages (rat, 1:100, anti-CD11b; MCA7; Serotec, Raleigh, USA) was performed following standard methods using an avidin-biotin system (Vector Laboratories, Burlingame, USA) and 0.02% diaminobenzidine as chromogen (Kem-En-Tec Diagnostics, Taastrup, Denmark). Negative controls included omission of either the primary or secondary antibody and gave no signals (not shown). To detect degenerating neurons, Fluoro Jade B (Millipore AG310) was used. The 10-μm thin brain cryosections mounted on slides were immersed in a solution containing 1% sodium hydroxide in 80% alcohol followed by 70% alcohol and water. They were then transferred to a solution of 0.06% potassium permanganate for 10 minutes and rinsed in water. After 20 min staining with a concentration of 0.0004% Fluoro Jade B, the slides were rinsed with water and then dried at approximately 50°C.

**Figure 6:**
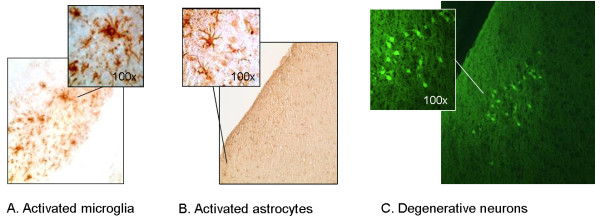
**Weight drop injury leads to activation of microglia (A.), astrocytes (B.) and neurodegeneration (C.) as shown by immunhistological stainings**.

**Figure 7:**
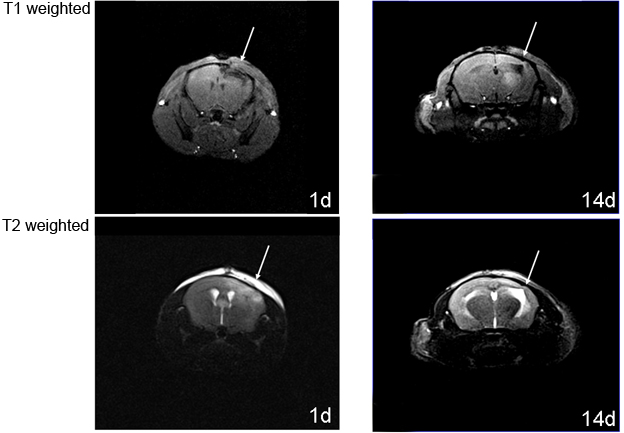
**Coronal T1 and T2 weighted magnetic resonance scans obtained on 1 day demonstrates a contusion in the ipsilateral hemisphere and on day 14 after injury enlarged ventricles indicating brain atrophy**.

One week after weight-drop injury we observed numerous activated microglia (cell bodies with numerous branching processes) (Figure 6) and astrocytes (a process that is accompanied by hypertrophy and increased expression of the glial-specific intermediate filament GFAP). At the same time, also degenerating neurons were detected after trauma (Figure 7).

#### 2.2.3 Magnetic resonance imaging

Morphological changes and progression of brain damage induced by weight drop can be assessed by magnetic resonance imaging (MRI) [29]. The representative T1- and T2-weighted MR-images in Figure 7, were obtained with a 3.0- Tesla magnetic resonance apparatus (Vision, Siemens) using a brain coil for rodents 1 day and 14 days after induction of injury. We detected a contusion in the ipsilateral hemisphere the 1st day after injury, and on the 14th day, enlarged ventricles were observed, indicating brain atrophy.

## 3 Outlook

In this article we provide a thorough methodological description of a straightforward weight-drop mouse model reproducing brain injuries similar to those experienced in the human with a mix of diffuse injury pattern and focal contusion. A major advantage of this model is that it allows neurological scoring immediately after injury and in the sequel. Thus, although the inflicted brain injury varies in its severity, clinically relevant randomization of mice into the various treatment groups is possible and this animal model might facilitate the discovery of improved drug treatments for TBI.
